# Metabolic Dialogue Shapes Immune Response in the Tumor Microenvironment

**DOI:** 10.1002/eji.202451102

**Published:** 2025-04-14

**Authors:** Fengxia Gao, Rushil Shah, Gang Xin, Ruoning Wang

**Affiliations:** ^1^ Department of Microbial Infection and Immunity Pelotonia Institute for Immuno‐Oncology The Ohio State University Columbus Ohio USA; ^2^ Center for Childhood Cancer Research Hematology/Oncology & BMT Abigail Wexner Research Institute at Nationwide Children's Hospital Department of Pediatrics The Ohio State University Columbus Ohio USA

## Abstract

The fate of immune cells is fundamentally linked to their metabolic program, which is also influenced by the metabolic landscape of their environment. The tumor microenvironment represents a unique system for intercellular metabolic interactions, where tumor‐derived metabolites suppress effector CD8^+^ T cells and promote tumor‐promoting macrophages, reinforcing an immune‐suppressive niche. This review will discuss recent advancements in metabolism research, exploring the interplay between various metabolites and their effects on immune cells within the tumor microenvironment.

## Graphical abstract

Metabolic antagonism in the TME suppresses CD8^+^ T cell function by depleting essential nutrients and generating toxic byproducts. Metabolic symbiosis between tumor cells and TAMs fosters an immunosuppressive microenvironment. Disrupting metabolic interactions can overcome immune suppression, boost antitumor immunity, and enhance immunotherapy efficacy.

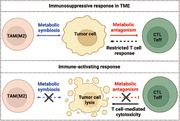

## 1 Introduction

### 1.1 Intercellular Metabolic Interaction

Metabolites have been known to serve as an additional form of intercellular communication outside of soluble factors (e.g., proteins, nucleotides, and vesicles) and direct effects of cell–cell contact and are vital to support the cell's physiological functions. For cells in highly metabolic tissues with high energy demands, like the brain, there is a sophisticated intercellular shuttle system involving glucose, glutamine, pyruvate, and lactate between neurons and astrocytes to meet physiological demands [1, 2, 3, 4]. Similar metabolic coordination is observed in the retina between glial cells and photoreceptor cells and in the testis between Sertoli cells and germ cells [5, 6]. Emerging evidence has shown that various pathogen‐derived metabolites mediate intercellular host–pathogen interactions, critically impacting homeostasis during infections [7, 8]. The tumor microenvironment (TME) represents a unique niche of metabolic derangement, where tumor‐surrounding cells can support or restrict tumor cell growth in part through competition for nutrients or metabolic symbiosis, contributing to altered immune responses as a result of metabolic reprogramming [9, 10, 11].

### 1.2 Tumor Immunity

As the primary component of antitumor immunity, tumor antigen‐specific cytotoxic T (CTL) execute their tumor‐killing activity through multiple means, including the secretion of effector cytokines such as IFN‐γ or soluble factors (perforin and granzyme B) to disturb targeted cell proliferation and induce tumor cell lysis [12]. Mounting evidence indicates that strengthening the amplitude and quality of T cell response is one of the most promising approaches to enhance therapeutic antitumor immunity [13, 14, 15]. In addition to T cells, macrophages represent a critical arm of innate immune responses and frequently dictate immunity against tumors [16]. Within advanced‐stage tumors, tumor‐associated macrophages (TAMs) can release various growth factors and cytokines that support tumor cell survival and proliferation while inhibiting antitumor immunity [17]. Both antitumor T cells and protumor activity of TAMs depend on various environmental cues, including metabolites. Given the limited nutrient availability within the tumor microenvironment, the high consumption of these nutrients by tumor cells may create a metabolic barrier for intratumoral T cells while promoting the formation of immunosuppressive TAMs [9, 18].

### 1.3 Metabolic Reprogramming in TEM

The “Warburg effect” is a hallmark of malignancy, characterized by the enhanced glycolytic capacity of cancer cells even in oxygen‐rich conditions (aerobic glycolysis). To meet their demands for growth and proliferation, cancer cells engage in diverse metabolic modes, including aerobic glycolysis [19, 20, 21, 22]. Consequently, cancer cells with high metabolic activity significantly affect the TME, leading to nutrient depletion, hypoxia, acidity, and the production of toxic metabolites to immune cells [23, 24, 25]. Within the TME, a substantial portion of glucose undergoes aerobic glycolysis, generating high levels of lactate and protons (H^+^), which lower the intratumoral pH. The balance between lactate‐generating glycolysis and oxidative phosphorylation is likely influenced by the degree of hypoxia, which can vary widely and heterogeneously within the TME [26]. Notably, in moderately hypoxic regions, extracellular carbonic anhydrase enzymes convert carbon dioxide (CO_2_) from mitochondrial respiration into HCO_3_^−^ and H^+^ [27]. As a result, the tumor‐driven nutrient depletion and metabolic byproduct production impede the metabolic reprogramming of immune cells, particularly CD8^+^ T cells and macrophages, within the TME [10].

T cells play an important role in the host response against malignant cells. In response to the emergence of malignant tissue, cancer‐reactive CD8^+^ T cells are recruited to the tumor to provide antigen‐specific responses to suppress tumor growth. The distinct metabolic profiles of T cells are intimately linked to their status and function [28]. Upon activation, CD8^+^ T cells signal through c‐Myc and MAPK pathways to increase aerobic glycolysis and glutaminolysis and support their effector functions [28, 29, 30]. This rewired metabolic program makes effector T cells more vulnerable to TME‐mediated challenges (reduced oxygenation, nutrient deprivation, and lactate accumulation), leading to attenuated effector functions and exacerbated exhaustion [31]. Dysregulated metabolism is a key driver of maladaptive tumor‐reactive T lymphocytes within the tumor microenvironment [10, 32].

TAMs represent approximately 50% of immune cells within the tumor microenvironment, but most act as protumoral M2‐like macrophages [33]. Mounting evidence has shown that switching the TAMs phenotype from M2 to M1 may promote antitumor activity, implicating a phenotypic plasticity of TAMs [34, 35, 36]. M1 macrophages, characterized by high MHC II, CD80, and CD86 expression, secrete pro‐inflammatory cytokines IL‐12 and TNF‐α, whereas M2 macrophages express CD163, CD206, and Arg1 and produce IL‐10, fostering an immunosuppressive TME. As functionally plastic cells, macrophages tightly regulate their metabolic programs to support functional properties. M1 macrophages engage in glycolysis to produce lactate and convert arginine to nitric oxide (NO) and citrulline to support antimicrobial and antitumor ability [37]. In contrast, M2 macrophages use fatty acid oxidation (FAO) and the tricarboxylic acid (TCA) cycle to produce energy and building blocks, meanwhile metabolizing tryptophan into kynurenine and converting arginine to ornithine and polyamines, which contribute to immune suppression and tumor escape [38, 39, 40].

### 1.4 Intercellular Metabolic Interaction

**FIGURE 1:**
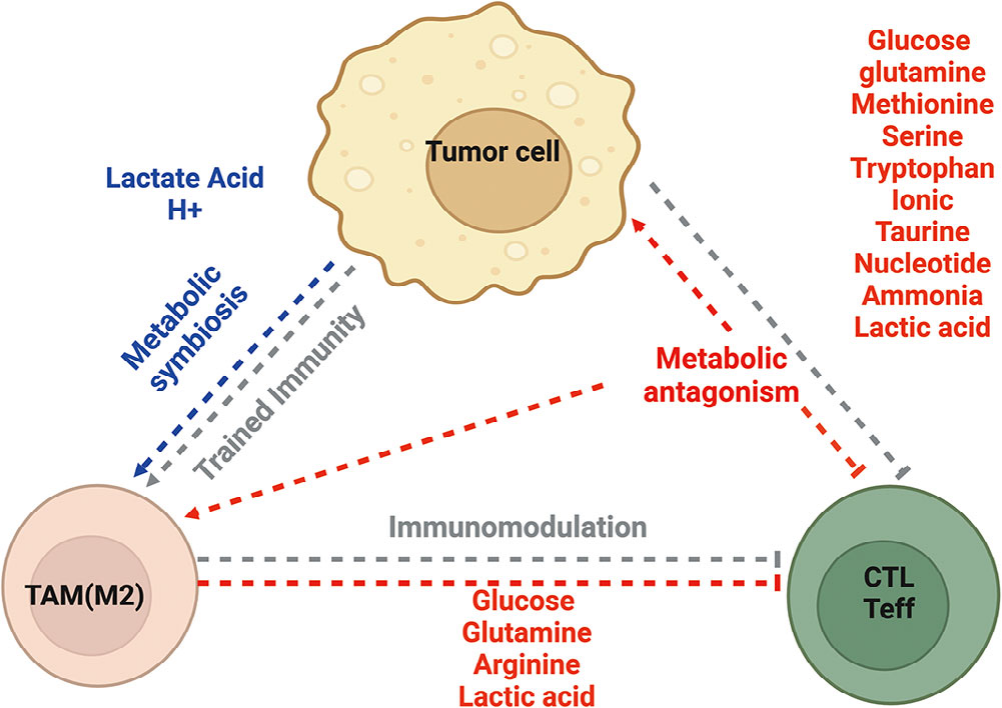
Metabolic interplay among tumor cells, CD8^+^ T cells, and TAMs. Shuttling of metabolites among different cell compartments can act as a mechanism for intercellular communication and coordination in TME. Tumor cells, CD8^+^ T cells, and TAMs may engage in potential metabolic antagonism (highlighted in red) with each other. This antagonism arises from competition for key nutrients and metabolic pathways. Conversely, TAM‐M2 cells may preferentially utilize metabolic byproducts of tumor cells, forming a potential metabolic symbiosis (highlighted in blue) that supports tumor growth and immune evasion.

The interaction between the immune system and the tumor within TEM is a complex and dynamic process [41]. Aerobic glycolysis and glutaminolysis are the dominant cancer metabolic pathways [42, 43]. Such heightened glucose and glutamine consumption often results in the depletion of oxygen and nutrients (glucose, glutamine, etc.), in addition to accumulating metabolic end‐ or by‐products (lactate, proton, ammonia, etc.) in the tumor microenvironment [41, 44]. Upon activation, CD8^+^ T cells shift from OXPHOS to glycolysis to support the rapid proliferation and production of cytokines necessary for effective antitumor activity [29, 30]. However, tumors consume large amounts of glucose and other nutrients, leading to competition between cancer cells and T cells/macrophages. Coincidentally, metabolic products of tumors were preferentially utilized by TAMs, forming a potential metabolic symbiosis in the tumor microenvironment, which will contribute to the immunosuppressive microenvironment (Figure 1).

### 1.5 Metabolic Antagonisms

#### 1.5.1 Glucose and Glutamine

Glucose and glutamine are the primary nutrients for cancer cells, T cells, and macrophages, which share similar metabolic profiles. As a result, this leads to fierce competition for the limited supply of glucose and glutamine in TME. The capacity to uptake these essential nutrients is vital for the survival and function of different cell subsets in the TME. One recent PET imaging study revealed that intratumor myeloid cells (including F4/80+ TAMs) possess the most significant capacity to uptake glucose within TME, followed by T cells and cancer cells [45]. Consistent with this observation, histological analysis showed that glucose transporter 1 (GLUT1) is highly expressed in TAMs localized in hypoxic regions of both transplanted and autochthonous murine breast tumor tissues [46]. In contrast, cancer cells exhibited the highest uptake of glutamine across a range of cancer models [45]. They used 2‐NBDG, a fluorescent glucose analog, to measure glucose uptake in different cell populations. Additionally, the study assessed GLUT1 expression to quantify glucose transport activity. This distinct nutrient utilization profile was programmed in a cell‐intrinsic manner through mTORC1 signaling and the overexpression of genes related to glutaminolysis, such as GLS1 and GLUT1 [47]. In addition, tumor cells upregulate glutamine transporters, such as solute carrier family 1 member 5 (SLC1A5) and glutaminase (GLS), to enhance glutamine uptake and utilization [48].

The conflict between nutrient utilization leads to metabolic stress on both tumor and immune cells. However, the metabolic flexibility of cancer allows them to survive under low glucose and glutamine conditions [49]. Furthermore, the production of lactate and CO2 from glycolysis and glutaminolysis results in the acidification of the tumor microenvironment, which favors the development of more aggressive and invasive tumor cells [50, 51]. Distinct from cancer cells, TAMs possess a superior capacity to take up glucose, allowing them to outcompete other cell types within the tumor, promoting their pro‐tumor and immunosuppressive function [10, 52, 53]. Supporting these findings, a recent study using an orthotopic murine pancreatic ductal adenocarcinoma (PDAC) tumor model demonstrates that the GLUT1 depletion in TAMs via lysozyme M‐cre (LysMcre) improved natural killer and CD8^+^ T cell activity and inhibited tumor progression. In contrast to tumor cells and TAMs, effector T cells are deprived of glucose and glutamine, which are crucial for their effector functions [54, 55] and survival [56, 57, 58]. These findings suggest that the metabolic interaction, particularly the competition for limited nutrients in the tumor microenvironment, can profoundly impact antitumor immunity.

#### 1.5.2 Methionine

The catabolism of the essential amino acid methionine has been reported as a biomarker of tumors in various studies [59]. In cancer cells, methionine is primarily converted into S‐adenosylmethionine (SAM) and polyamines. SAM serves as a critical methyl donor in the methylation of deoxyribonucleic acid (DNA), ribonucleic acid (RNA), proteins, and lipids. Aberrant methylation patterns, such as hypermethylation of tumor suppressor genes (like p53) and hypomethylation of oncogenes, are common phenomena in cancer and contribute to tumorigenesis [60]. Increased methionine catabolism in tumor cells resulted in methionine depletion in the extracellular space. T cells are reliant on amino acid transporters, including Slc7a5, for methionine transport [61]. Mechanistically, Slc7a5‐null T cells lack effective engagement with antigen as well as have an impaired ability to engage in inflammatory profiles and cellular cycling profiles1. Slc7a5‐null T cells are dramatically lower in methionine levels and subsequently have reductions within mTORC1 activation, c‐MYC expression, as well as reduced glycolysis and engagement in central carbon metabolism [62]. In addition to this, deprivation of methionine can suppress T effector function by causing lower intracellular levels of methionine and SAM and resulting in loss of demethylation at lysine 79 of histone H3 (H3K79me2) [63]. The loss of H3K79me2 leads to low expression of STAT5 and impaired T‐cell immunity, which, in turn, suppresses antitumor‐immune responses [63]. Conversely, the conversion of methionine to polyamines is essential for cancer cell proliferation but also plays a broad role in modulating immunity [63]. The extracellular accumulation of polyamines alters the expression of T cell surface receptors, including T cell receptor (TCR) and co‐stimulatory molecules like CD28, which directly impact the signaling pathways necessary for T cell proliferation and cytokine production [64]. Polyamines can also influence the expression of immune checkpoint molecules, such as programmed death‐ligand 1 (PD‐L1) on tumor cells. Increased PD‐L1 expression inhibits T cell activity through PD1 engagement, contributing to immune evasion by the tumor [65]. Additionally, polyamines promote the polarization of macrophages toward the immunosuppressive M2 phenotype, which promotes tumor growth and inhibits immune responses [66]. Mechanistically, polyamine biosynthesis regulates the activity of the enzyme eIF5A through a posttranslational modification process referred to as hypusination. The hypusinated form of eIF5A facilitates the efficient expression of numerous mitochondrial proteins that enhance OXPHOS and alternative macrophage activation [67]. The polyamine compound spermidine can stimulate the expression of immunosuppressive factors like arginase and inhibit the pro‐inflammatory activities of M1 macrophages by decreasing the production of cytokines and the expression of costimulatory molecules CD80 and CD86 [66]. In summary, methionine depletion and polyamine accumulation cooperatively create an immunosuppressive microenvironment in tumors.

#### 1.5.3 Serine

Extracellular serine is essential for optimal T cell expansion and effector function. T cells require serine to fuel one‐carbon metabolism to support their proliferation. T cells rely on serine to maintain effector functionality. Activated T cells increase the expression of amino acid transporters such as ACT2 to increase the extracellular uptake of serine [68, 69]. When deprived, T cells can generate serine de novo from glycine and other one‐carbon metabolites to rescue deprivation. Notably, low levels of extracellular serine compromise de novo purine nucleotide synthesis and cellular division [69]. While T and tumor cells can produce serine, the de novo serine synthesis capacity is often suppressed in the TME. Consequently, the competition between tumor and T cells for limited extracellular serine can lead to the suppressed T cell proliferation. In contrast, TME upregulates the phosphoserine aminotransferase 1 (PSAT1) and promotes serine biosynthesis in TAMs by activating the protein kinase RNA‐like ER kinase (PERK) signal pathway [70, 71]. This enhanced serine metabolism supports mitochondrial fitness and provides ketoglutarate, which enables jumonji domain‐containing protein 3 demethylase‐dependent epigenetic modification that amplifies the immunosuppressive function in intratumoral macrophages. These activated TAMs can lead to further mechanistic suppression of T cells by releasing reactive oxygen species and other intermediates, which promote lipid oxidation, further creating a harsh metabolic environment [72]. Collectively, these studies indicate the importance of serine metabolism in the interplay between tumor cells, T cells, and TAMs, thereby influencing the balance of antitumor immunity and tumor progression.

#### 1.5.4 Tryptophan

Tryptophan is an essential amino acid reported as a tumor biomarker in various studies [73]. In tumor cells, tryptophan is converted to kynurenine by indoleamine‐2,3‐dioxygenase (IDO) and tryptophan‐2,3‐dioxygenase (TDO). High expression of IDO and TDO in various cancers facilitates tumor cells in evading immune surveillance [74, 75]. Upregulated tryptophan catabolism in tumor tissue resulted in the depletion of tryptophan in the extracellular space, which may cause dysfunction and apoptosis of effector T cells [76, 77]. The accumulation of kynurenine and its downstream metabolites, including 3‐hydroxykynurenine, can induce T cell apoptosis and promote the expansion of regulatory T cells (Tregs), thereby further suppressing the antitumor immunity [73, 78]. Moreover, kynurenine and potentially other catabolic metabolites of tryptophan are identified as natural ligands of aryl hydrocarbon receptor (AhR), which plays a broad role in modulating immunity [78]. The AhR activity is elevated in TAMs and plays a vital role in macrophage polarization [79]. Deletion of AhR in TAMs or pharmacologic inhibition of AhR reduced PDAC tumor growth, improved efficacy of immune checkpoint blockade, and increased intratumoral frequencies of IFN‐γ^+^ CD8^+^ T cells [39]. Consistent with this observation, high AhR expression is associated with rapid disease progression and mortality, as well as with an immunosuppressive TAM phenotype in patients with PDAC [39]. Thus, tryptophan depletion and its catabolite accumulation cooperatively foster an immunosuppressive microenvironment in tumors.

#### 1.5.5 pH and ion

Lactate produced by tumor cells causes acidification of the TME [80]. This acidification helps tumor cells invade surrounding tissues by promoting the activity of proteases, such as matrix metalloproteinases (MMPs), which degrade the extracellular matrix [81]. The acidic environment can also lead to the selection of more aggressive cancer cell phenotypes and promote drug resistance [82]. The acidic microenvironment can impede the activity of immune cells such as T cells and natural killer (NK) cells. In addition, low pH conditions can impair the function of dendritic cells, which are crucial for antigen presentation and the activation of T cells. Proton pumps, such as V‐ATPases, and proton exchangers, like sodium‐hydrogen exchanger 1 (NHE1), are often upregulated in tumors and are potential therapeutic targets. Inhibitors of these proton pumps can disrupt the acidic microenvironment and inhibit tumor growth. Similarly, proton exchange is regulated by other cell membrane channels and transporters, such as Na^+^/H^+^ exchangers (NHE), Na^+^/H^+^ ATPase, and epithelial sodium channels (ENaC), which are often dysregulated in cancer cells. For example, increased intracellular sodium levels can promote cancer cell proliferation and migration. The Na^+^/H^+^ exchanger (NHE1) plays a role in regulating intracellular pH and cell volume. Necrotic tumor cells can release intracellular potassium [K^+^] ions into the extracellular fluid and cause profound suppression of T cell effector function. Potassium‐mediated immune suppression is mediated through the serine/threonine phosphatase protein phosphatase 2‐dependent suppression of Akt‐mTOR signaling [83]. Accordingly, augmenting potassium efflux in tumor‐specific T cells by overexpressing the potassium channel Kv1.3 lowers [K^+^], improves effector functions *in vitro* and *in vivo*, and enhances tumor clearance and survival in melanoma‐bearing mice [83]. In addition, the acidic pH of 6.1 can improve the expression of protumor molecules such as arginase 1 (ARG1) in macrophages [84]. The acidification of TME can be sensed by macrophages by a G protein‐coupled receptor (GPCR), such as GPCR64 and GPCR64 [85, 86]. Activating this signaling pathway enhances the production of cyclic AMP (cAMP) to upregulate the expression of Inducible cAMP Early Repressor (ICER) and other genes associated with immunosuppressive macrophages, like Arg1, Clec10a, and Vegfa [86]. Neutralization of intratumoral acidity can delay tumor progression by inhibiting protumor differentiation of TAMs [87].

#### 1.5.6 Taurine

Taurine, an amino acid derivative, is primarily synthesized in the liver through cysteine and methionine metabolic pathways. In tumors, taurine is mainly transported by solute carrier family 6 member 6 (SLC6A6), and its level correlates with aggressiveness and outcomes in multiple cancers [88]. Tumor cells outcompete CD8^+^ T cells for taurine by overexpressing SLC6A6 [29, 88], which consequently induces endoplasmic reticulum (ER) stress in CD8^+^ T cells through a PERK‐ATF4‐dependent mechanism [88]. Activating transcription factor 4 (ATF4) transactivates multiple immune inhibitory genes and induces T cell death, malfunction, and exhaustion [88]. In contrast, culturing CD8^+^ T cells with taurine *in vitro* enhances their secretion of cytotoxic cytokines, PLCγ1‐mediated calcium production, and MAPK signaling, which determine the functional state of CD8^+^ T cells and the therapeutic efficacy of programmed cell death protein 1 (PD‐1) blockade antibodies in tumors [89]. Consistent with the preclinical studies, high serum taurine levels in lung cancer patients correlate with high levels of cytotoxic cytokines (IFN‐γ and TNF‐α) and better outcomes in responding to PD‐1 blockade therapy [89]. Furthermore, TAMs can produce taurine to support the growth of prostate cancer cells by signaling through the Liver X receptor alpha/stearoyl‐coenzyme A desaturase 1 (LXR‐α/SCD1) pathway to protect them from ferroptosis [90]. This tumor cell‐macrophage interaction can be abolished by inhibiting the taurine transporter, TauT, thereby sensitizing the cancer cells to ferroptosis [90].

#### 1.5.7 Nucleotide and Nucleoside Metabolism

Nucleotides, the building blocks of DNA and RNA, are intrinsically required for driving cancer growth [91]. In addition, their catabolic products (nucleosides) can directly serve as alternative carbon sources to support the proliferation of cancer and immune cells under nutrient‐scarce conditions. The ribose moiety in inosine, uridine, and thymidine has been reported to provide carbon to sustain cell proliferation [91, 92, 93, 94]. Within the TME, cancer cells can actively excrete nucleotides through exocytosis and transporters [95, 96, 97]. Furthermore, hypoxia and cytotoxic reagents can induce nucleotide release by damaging cancer cells [97, 98, 99]. Apart from tumor cells, immune cells, such as macrophages and T cells, can also release nucleotides. For TAMs, release pyrimidines such as deoxycytidine, modulating chemotherapy resistance [100]. Additionally, macrophages undergoing necrotic cell death passively release UDP activating P2Y6 receptors and inducing MCP‐3 expression. Effector CD8^+^ T cells, under hypoxia or cytotoxic stress, can undergo activation‐induced cell death, releasing ATP via P2×7 receptors, while necrotic T cells passively release nucleotide [101].

Both tumor and immune cells express ecto‐nucleotidases, which are critical in controlling nucleotides and nucleoside turnover in the TME [102]. It is well‐established that purine nucleosides can engage purinergic receptors in autocrine or paracrine manners, fostering an immune suppressive microenvironment [103]. Recent studies have also shown that pyrimidine nucleosides are critically involved in mediating communications between macrophages and tumor cells through engaging cytidine deaminase (CDA) and P2Y6 signaling [100]. Moreover, CDA expression could predict PDAC patient outcomes in response to immune check point (ICB) therapy [104]. Thus, nucleotide metabolism in TAMs significantly influences their function and promotes tumor progression.

#### 1.5.8 Ammonia

Tumor cells, due to increased amino acid catabolism, produce a large amount of ammonia (NH_3_). Ammonia is a weak base that generates ammonium ions (NH4^+^) when combined with hydrogen ions (H^+^). This allows ammonia to buffer acidic changes in the microenvironment and regulate pH, helping tumor cells adapt to harsh conditions and improve their survival and proliferation. Recent studies have shown that imbalances in intracellular ammonia in the TME can suppress TCR signaling and inhibit T cell activation, thereby reducing T cell proliferation and cytokine secretion, such as IL‐2 and IFN‐γ [105, 106]. Interestingly, memory T cells have less ammonium present compared with effector CD8^+^ T cells and are more resistant to NH4Cl‐induced cell death [107]. Further investigation into memory CD8^+^ T cells revealed enhanced utilization of Carbamoyl phosphate synthetase 1 (CPS1) to form arginine in the cytosol [107], which can translocate to the mitochondria, catalyzed by arginase 2 and NO synthase, and produce urea, ornithine, NO, or citrulline. CPS1 ablation compromised the memory cell differentiation, implying a role in the balance of ammonia and utilization of the urea cycle in altering CD8^+^ T responses [107]. Other pathways, including the malate shuttle enzyme glutamic‐oxaloacetic transaminase 1 (GOT1), can also scavenge ammonia by converting 2‐KG to glutamate [108]. Deletion of GOT1 compromised effector function, such as IFN‐γ and TNF cytokine production, in CD8^+^ T cells, and accelerated their dysfunctional process [108]. Therefore, targeting and balancing ammonia concentration could dictate the short‐term and sustained response of effector T cells in the TME. In metastatic mouse models of colon cancer, tumors impair CD8^+^ T cell functionality through ammonia production and accumulation in the TME [105]. Therapeutically, an imbalance of ammonia can hinder the efficacy of PD‐L1 therapy and decrease tumor‐associated ammonia‐enhanced immune checkpoint efficacy and long‐term durability of CD8^+^ memory responses.

#### 1.5.9 Lactate

Tumor cells actively engage in aerobic or anaerobic glycolysis to convert glucose‐derived carbon into lactate. Recent studies have demonstrated that both tumor and immune cells can utilize and catabolize lactate [109, 110, 111, 112]. For instance, isotope tracing of carbon‐13 (13C) labeled lactate in non‐small‐cell lung cancer (NSCLC) patients revealed extensive 13C labeling of TCA cycle intermediate metabolites within surgically removed tumor tissues [113]. This suggests lactate is an *in vivo* fuel for the TCA cycle and OXPHOS in cancer cells. Outside of the cancer cell‐intrinsic fates of lactate, lactate can also be excreted into the TME as an immunosuppressive acidic factor. Lactic acid is a weak acid that dissociates in the TME, reducing the pH and possibly suppressing immune functionality simply due to pH changes [110, 111, 114].

Studies have shown that acidification of the medium by physiologically relevant lactate levels partially decreased IFN‐γ and IL‐2 production in T cells [110, 111]. Buffering the culture media back to physiological pH (7.4) restores cytokine production in human CTLs *in vitro* [110, 115, 116]. In addition to the pH‐dependent changes in functionality, tumor‐derived lactate can significantly disrupt T‐cell metabolism [117]. Tumor‐derived lactate can induce a pyruvate utilization switch in CD8^+^ T cells, enhancing pyruvate carboxylase (PC) utilization instead of pyruvate dehydrogenase (PDH). Eventually, this results in a negative feedback loop that impairs the TCA cycle and reduces substantial levels of intracellular succinate within T cells [115]. Lack of succinate production failed to induce specific G protein‐coupled receptor succinate receptor 1 (SUCNR1) signaling, thus compromising T cell responses [115]. SUNCR1 activation is essential for CD8^+^ killing capacity as well as IFN‐γ production. Accordingly, inhibiting PDH synergized with T cell responses in a SUNCR1‐dependent manner in a high lactate setting since SUNCR1 KO T cells failed to mount antitumor responses when PDH was inhibited [115, 117].

In the TME, both CD8^+^ T cells and Tregs utilize monocarboxylate transporters (MCTs) to regulate lactate metabolism but in distinct ways. MCT1, a bidirectional lactate transporter, is primarily involved in lactate uptake in both cell types, whereas MCT4 mainly facilitates lactate export. Effector CD8^+^ T cells predominantly express MCT1, particularly under glucose‐deprived conditions, enabling them to absorb extracellular lactate to sustain metabolic activity [118]. However, excessive lactate uptake via MCT1 can impair CD8^+^ T cell function, leading to mitochondrial dysfunction, metabolic reprogramming, and reduced IFN‐γ and IL‐2 production [110]. Notably, PD‐1^+^CD8^+^ T cells exhibit higher MCT1 expression, making them more susceptible to lactate‐induced metabolic suppression [119]. While CD8^+^ T cells can upregulate MCT4 upon activation or hypoxia to expel intracellular lactate, MCT4 plays a relatively minor role compared with MCT1‐mediated lactate uptake. In addition, new studies have found that exhausted T cells depend on lactic acid uptake through monocarboxylate transporter 11 (MCT11) to reinforce an exhausted phenotype [120]. Genetic ablation and pharmacological blockade of MCT11 or Slc16a11 lead to dramatic reductions in an exhausted population and improvements in ICB responsiveness in preclinical models [120]. Regarding exhaustion, lactic acid uptake through MCT11 leads to reduced stem‐like signatures of T cells and enhanced genes associated with terminal exhaustion, including Havcr2 (TIM3), Eomes, and Id2, and disrupted mitochondrial biogenesis [120].

In contrast, Tregs exhibit a metabolic profile that favors lactate utilization rather than export. Intratumoral Tregs show high MCT1 expression, allowing efficient lactate uptake, which supports Foxp3‐mediated metabolic reprogramming and enhances their suppressive function [112]. MCT1‐deficient Tregs exhibit impaired lactate uptake, resulting in reduced suppressive function and enhanced antitumor immunity. Unlike CD8^+^ T cells, Tregs express relatively low levels of MCT4, suggesting that lactate import rather than export is crucial for their function in the TME. Furthermore, MCT1‐mediated lactate uptake has been linked to PD‐1 upregulation in Tregs via NFAT1 nuclear translocation, reinforcing their immunosuppressive activity. Notably, intratumoral Tregs exhibit higher PD‐1 expression in highly glycolytic tumors, such as MYC‐amplified and liver tumors, and PD‐1 blockade paradoxically enhances Treg function, contributing to immune checkpoint therapy resistance [119]. Additionally, Wagner et al. demonstrate that lactate can induce PD‐1 expression on innate lymphoid cells (ILCs), further contributing to immunosuppression in the TME [121]. Beyond T cells, lactate also plays a role in modulating other immune cell populations in the TME. This suggests that lactate‐driven PD‐1 upregulation is not exclusive to CD8^+^ T cells and Tregs but extends to other immune subsets, highlighting lactate as a central regulator of immune suppression in the tumor context. Collectively, these findings underscore the critical role of MCT1 in Treg‐mediated immune suppression and suggest that targeting MCT1 may represent a promising strategy to selectively disrupt Treg function and enhance antitumor immunity in the TME.

In contrast to the above findings, activating CD8^+^ T cells with high amounts of lactate is also reported to enhance immunotherapy efficacy. CD8^+^ T cells primed with high amounts of lactate before infusion displayed several markers associated with a stem‐like memory fate, including higher amounts of TCF1 and enhanced tumor control [122]. Lactate supplementation was also found to improve accessibility to TCF1 within CD8^+^ T cells and the efficacy of immune checkpoint therapy [122]. Taken together, the effects of intratumoral lactate are substantial, but the context dictates the impact of CD8^+^ T cell functionality.

Elevated lactate levels in the TME significantly enhance the ability of macrophages to promote tumor growth and suppress antitumor immunity. Specifically, lactate induces the upregulation of hypoxia‐inducible factor 1α (HIF1α), which leads to increased expression of vascular endothelial growth factor (VEGF) and arginase 1 (Arg1) in TAMs [123]. Furthermore, lactate also modulates the interplay between metabolism and epigenetics, favoring an M2‐like macrophage polarization within the tumor [124]. Isotope tracing studies have demonstrated that macrophages can metabolize extracellular lactate to fuel the TCA cycle and produce citrate [124]. This lactate‐derived citrate is then cleaved by ATP citrate lyase, generating acetyl‐coenzyme A, which serves as a critical donor for histone acetyltransferases, thereby increasing histone acetylation. Functional assays suggest that this ACLY‐dependent histone acetylation is enriched at the promoter regions of M2‐specific immunosuppressive genes, ultimately leading to an attenuated T‐cell response and accelerated tumor progression.

### 1.6 Metabolic Symbiosis

The interplay of metabolic byproducts and intermediates within the TME can lead to radically different outcomes regarding immune responses and immunotherapy efficacy. The complex milieu of factors within the TME can evolve over tumor progression to create a more hostile environment for immune cells. Heterogeneity in the TME can lead to the preferential growth and differentiation of various immune cell populations and other cells, including critical populations like fibroblasts, during their transition to cancer‐associated fibroblasts [125]. Fibroblasts play a crucial role in fostering an immune‐suppressive TME in solid tumors. Within immune‐excluded sarcoma, highly glycolytic cancer‐associated fibroblasts (glyCAF) preferentially expand and accumulate, which craft a hostile environment for immune cells. The high glucose transporters (GLUT1) expression in these glyCAFs deprives the nutrients needed for effector T cell responses [125]. Thus, the blockade of GLUT1 on glyCAFs enhanced glucose bioavailability, enhancing T‐cell responses [125]. Furthermore, cancer cells take up other critical nutrients like glutamine and serine more efficiently than immune cells through changes in the protein expression of corresponding transporters. In addition, cancer cells also increase the expression of glutamine transporters such as SLC1A5, further competing with immune cells for nutrients. As tumors progress, the accumulation of TAMs begins to modify the metabolic landscape in TME through impacting the fibrotic cells [126]. This interaction can induce a collagen biosynthesis program that synergizes with transforming growth factor beta (TGF‐β) signaling in the TME to consume arginine and synthesize proline [126]. Collectively, the sequestration of arginine and the accumulation of proline and polyamines impair T‐cell responses and compromise antitumor immunity. Other metabolites can also interplay with one another in the cancer ecosystem to suppress immune responses, such as lactate.

**FIGURE 2:**
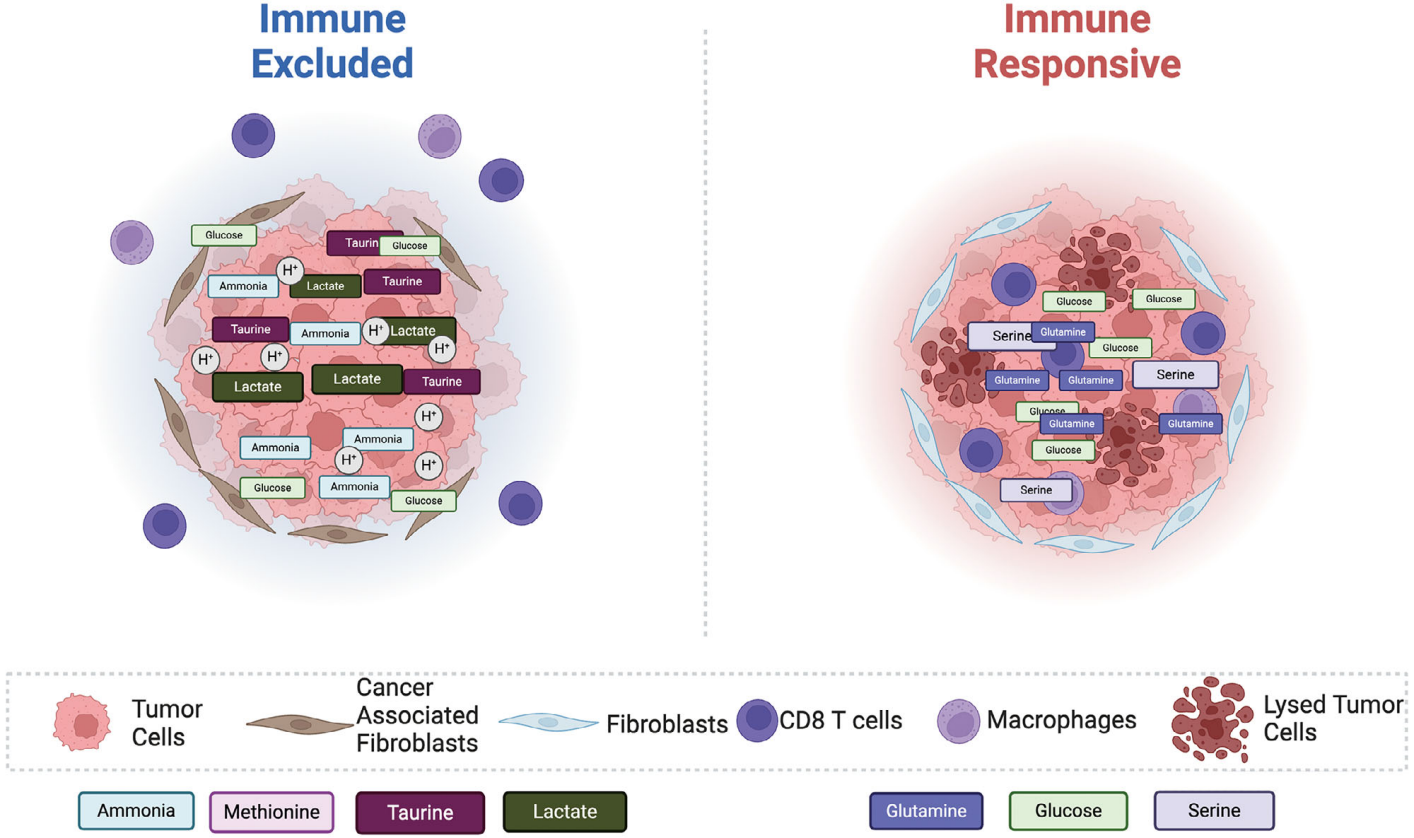
Heterogeneity in metabolite concentrations is apparent within immune‐responsive and excluded tumors. Immune‐excluded tumors can accumulate intermediates toxic for CD8^+^ T cells and TAMs, such as taurine, ammonia, and lactate, compromising antitumor functionality and promoting differentiation of cancer‐associated fibroblasts. Immune‐responsive tumors may provide more metabolic intermediates and nutrients necessary for antitumor functions, such as serine, glucose, and glutamine.

Tregs can take up lactate rapidly and catabolize it through the TCA cycle [112]. Moreover, supplemental lactate within the culture medium enhanced Tregs differentiation [127]. Lactate‐treated human Tregs express elevated levels of PD‐1 and display a heightened ability to inhibit the proliferation of effector T cells [119]. Additionally, lactate present in the TME may also help to foster an immunosuppressive environment by modulating macrophage differentiation and functions. It has been shown that IL‐4‐induced anti‐inflammatory macrophages can also utilize lactate through the TCA cycle [124]. In TAMs, lactate can induce the expression of VEGF and Arg1 through transcription factor HIF1α [128, 129]. Furthermore, lactate may regulate macrophage gene expression through epigenetic mechanisms since lactate treatment can enhance the acetylation of histone H3 at lysine 9 in the promoter regions of Arg1 [124]. Collectively, tumor‐derived lactate and its catabolic metabolites may form a layer of metabolic symbiosis with TAMs and Tregs to promote tumor growth. These studies illuminate the need for new therapeutics that do not simply examine one immune population and its capacity to engage in antitumor functionality but rather the metabolic system that contributes to the efficacy of responses (Figures 1 and 2).

### 1.7 Future Directions and Therapeutic Perspectives

The metabolic antagonism and symbiosis between tumor and immune cells promote cancer progression and the infiltration of immunosuppressive TAMs and Tregs [87, 112, 119, 127]. Conversely, these interactions contribute to the functional inhibition of CD8^+^ T cells [110, 111, 130]. Understanding the nature of these metabolic interactions also provides opportunities to develop tailored therapeutics based on several aspects of the metabolic landscape in the TME. Understanding the intricate metabolic interactions in the TME presents an opportunity to develop tailored therapeutic strategies that target key metabolic vulnerabilities. The metabolic activity of CD8^+^ T cells is crucial for their effector function and is regulated by both intrinsic and extrinsic mechanisms. Intrinsically, TCR signaling and various TME‐derived factors contribute to a progressive decline in T cell metabolic fitness, ultimately impairing cytolytic activity [131, 132, 133, 134]. This metabolic suppression can be alleviated through ICB, such as antibodies targeting PD‐1. PD‐1 inhibition enhances glycolysis and mitochondrial function in CD8^+^ T cells, restoring their effector capabilities and reinvigorating antitumor immunity. This approach has revolutionized cancer immunotherapy, yielding significant and durable clinical responses across multiple malignancies. However, resistance remains a major challenge, often arising from extrinsic metabolic constraints within the TME. Nutrient depletion limits the energy and biosynthetic resources required for effective CD8^+^ T cell reactivation. While PD‐1 blockade enhances glycolytic capacity, persistent glucose restriction in the TME may still impair T cell function, reducing overall antitumor efficacy [135]. Additionally, although PD‐1 blockade can restore mitochondrial function, its effectiveness is contingent on the availability of key mitochondrial substrates, including amino acids, nucleotides, and fatty acids. To overcome these metabolic barriers and enhance ICB efficacy, further investigation is warranted into combinatorial strategies that integrate metabolic modulators or mitochondrial‐targeted therapies. Such approaches may help sustain CD8^+^ T cell function within the metabolically hostile TME, ultimately improving therapeutic outcomes and overcoming resistance to immune checkpoint blockade.

Some strategies could include increased cholesterol metabolism, tumor‐targeting of glutamine metabolism, altering the carbon sources of T cell metabolism, and genetic alterations of existing immunotherapy strategies. (1) Targeting cholesterol and lipids: Cholesterol metabolism plays a crucial role in regulating lymphocyte function, particularly CD8^+^ T cells, which rapidly reprogram through the SREBP and LXR transcription factors to enhance cholesterol biosynthesis and uptake while reducing efflux [136]. The genetic deletion of a cholesterol‐metabolizing enzyme, ACAT1, enhances the cytotoxic function of intratumoral CD8^+^ T cells. Furthermore, the adoptive transfer of ACAT1‐deficient CD8^+^ T cells into tumor‐bearing mice significantly delays tumor growth and improves the survival rate of the host mice. Moreover, linoleic acid is a significant positive regulator of CTL activity by improving metabolic fitness, preventing exhaustion, and stimulating a memory‐like phenotype with superior effector functions [137]. (2) Targeting glutamine metabolism: The metabolic competition for glutamine within the TME regulates antitumor immunity. The development of JHU083 [45, 138] and V‐9302 [139], selective inhibitors for tumor‐specific glutamine metabolism, addresses the “glutamine steal” scenario where cancer cells deprive lymphocytes of glutamine while enhancing T cell OXPHOS and anticancer immune responses in mice. (3) Targeting nucleosides: Adenosine (Ado) mediates immune suppression in the tumor microenvironment. By contrast, inosine (INO), the immediate metabolic product of Ado, could promote CD8^+^ T cell effector function under glucose‐restricted conditions [92]. Overexpression of Ado deaminase, which metabolizes Ado to INO, induced stemness and enhanced chimeric antigen receptor T‐cell (CAR‐T) functionality [140, 141, 142]. Similarly, treating CART cells with inosine leads to increased mitochondrial and glycolytic capacity, glutaminolysis, and polyamine synthesis, which reprograms the epigenome to enhance the stemness and function of T cells [140]. (4) Metabolically preconditioning CART cells: Ye et al. [143] developed a dead‐guide RNA (dgRNA)‐based CRISPR screen in activated CD8^+^ T cells and identified PRODH2 as gain‐of‐function targets for CAR‐T engineering. Targeted knock‐in or overexpression of PRODH2 improved CAR‐T cells’ metabolic and immune functions, enhancing CAR‐T‐based killing and *in vivo* efficacy in multiple cancer models. Under nutrient‐restricted conditions, IDH2 impedes the metabolic fitness of CART cells by restraining glucose utilization via the pentose phosphate pathway [143]. Through a mitochondria‐related compound screening, the FDA‐approved IDH2 inhibitor enasidenib enhances memory CART cell formation and sustains antileukemic cytotoxicity *in vivo* [143]. Beyond the genetic and pharmacological modulation of T cells, simple preconditioning of CAR‐T cells in defined conditions resembling metabolic fluctuations within the TME can also confer fitness and function benefits on T cells *in vivo* after transfer. Culturing CAR‐T cells *in vitro* under asparagine‐restricted, higher glucose, or high inosine conditioning media could improve their antitumor capacity after adoptive transfer into tumor‐bearing mice [85, 125, 126]. In the instance of asparagine restriction, reductions in asparagine within T cells can enable the expression of ATF4 and asparagine synthetase, which are hallmarks of the mitochondrial stress response. These transcriptional changes can revitalize T cells’ fitness within the TME [144]. Strategies like these could be coupled with ICB to further enhance the efficacy of metabolic‐driven immunotherapy strategies for ICB‐alone unresponsive tumors and in solid tumor settings with treatment of asparaginase [144, 145]. Thus, modulation or reprogramming of the altered metabolism of T cells represents a potential strategy to reinvigorate dysfunctional T cells for cancer treatment. In summary, our increasing understanding of the function and interplay based on metabolic antagonism symbiosis of cells is paving the way for more tailored manipulations of these cells *in vitro* and *in vivo* to enhance their efficacy against cancer.

## Conflicts of Interest

The authors declare no conflicts of interest.

## Data Availability

Data sharing does not apply to this article, as no datasets were generated or analyzed during the current study.
